# Ankle and toe muscle strength characteristics in runners with a history of medial tibial stress syndrome

**DOI:** 10.1186/s13047-017-0197-2

**Published:** 2017-04-11

**Authors:** Junya Saeki, Masatoshi Nakamura, Sayaka Nakao, Kosuke Fujita, Ko Yanase, Katsuyuki Morishita, Noriaki Ichihashi

**Affiliations:** 1 Human Health Sciences, Graduate School of Medicine, Kyoto University, 53 Shogoin-Kawahara-cho, Sakyo-ku, Kyoto, 606-8507 Japan; 2 Research Fellow of the Japan Society for the Promotion of Science, 5-3-1 Kojimachi, Chiyoda-ku, Tokyo, 102-0083 Japan; 3 Institute for Human Movement and Medical Sciences, Niigata University of Health and Welfare, Shimami-cho 1398, Kita-ku, Niigata, 950-3198 Japan; 4 Rehabilitation Group, Department of Medical Technique, Nagoya University Hospital, Tsurumai-cho 65, Syowa-ku, Nagoya, 466-0065 Japan

**Keywords:** Medial tibial stress syndrome, Shin splints, Toe flexor muscle strength, Hallux, Lesser toes, Metatarsophalangeal joint, Flexor hallucis longus, Flexor digitorum longus

## Abstract

**Background:**

A high proportion of flexor digitorum longus attachment is found at the posteromedial border of the tibia, which is the most common location of medial tibial stress syndrome (MTSS). Therefore, plantar flexion strength of the lesser toes could be related to MTSS; however, the relationship between MTSS and muscle strength of the hallux and lesser toes is not yet evaluated due to the lack of quantitative methods. This study investigated the muscle strength characteristics in runners with a history of MTSS by using a newly developed device to measure the muscle strength of the hallux, lesser toes, and ankle.

**Methods:**

This study comprised 27 collegiate male runner participants (20.0 ± 1.6 years, 172.1 ± 5.1 cm, 57.5 ± 4.0 kg). Maximal voluntary isometric contraction (MVIC) torque of the plantar flexion, dorsiflexion, inversion, and eversion of the ankle were measured by using an electric dynamometer. MVIC torque of the 1st metatarsophalangeal joint (MTPJ) and 2nd–5th MTPJ were measured by using a custom-made torque-measuring device. MVIC torques were compared between runners with and without a history of MTSS.

**Results:**

MVIC torque of the 1st MTPJ plantar flexion was significantly higher in runners with a history of MTSS than in those without it. In contrast, there were no significant differences in the MVIC torque values of the 2nd–5th MTPJ plantar flexion and each MVIC torque of the ankle between runners with and without a history of MTSS.

**Conclusion:**

A history of MTSS increased the isometric FHL strength.

## Background

Medial tibial stress syndrome (MTSS) is one of the most commonly observed injuries in runners [1, 2]. Previous studies have reported that 15.2% of high school runners developed MTSS during the 13 weeks of cross-country season [3] and 43.6% of high school runners developed MTSS during the 3 years of the follow-up period [4], and took 44–78 days to resume their original athletic level [5]. These studies indicated that prevention or treatment for MTSS is important; however, there is limited evidence regarding interventions being effective for the treatment and prevention of MTSS. A recent systematic review found that use of foot orthoses is beneficial for preventing MTSS [6]. Conversely, wearing thick-soled and heavy shoes decreased the running economy [7]. Thus, understanding of the etiology and correct intervention is important for preventing MTSS, without hampering the running performance.

A magnetic resonance imaging study has indicated that MTSS is a lesion in the junction of the periosteum and fascia [1]. A previous study reported that elongational stress of the lower limb muscles, such as the soleus, flexor digitorum longus (FDL), or tibialis posterior (TP) increased strain in the tibial fascia [8]. As larger navicular drop and excessive pronation during running are considered as risk factors of MTSS [9–11], it is assumed that MTSS is related to overuse of ankle inversion muscles. Furthermore, FDL had a higher proportion of attachment to the posteromedial border of the tibia, which is a more common location of MTSS than is the soleus (SOL) (FDL, 97%; SOL, 49%) [12], which suggested that MTSS could be developed by elongational stress of the FDL.

Considering the relationship between MTSS and ankle strength, a cross-sectional study reported that isokinetic inversion strength was relatively weaker than eversion strength of the ankle in patients with MTSS [13], and the mean number of standing heel-raise repetition test, an index of isotonic plantar flexion endurance of the ankle, was low [14]; however, a prospective study that investigated the risk factors of MTSS reported that preclinical ankle strength was not related to the development of MTSS [15], but the results conflicted with those of cross-sectional and prospective studies. The reason for this incongruence is assumed to be the inability by the participants to develop enough force due to pain at the time of measurement during the cross-sectional study. On the other hand, the participants did not have pain at the time of measurement during the prospective study.

In addition, the inversion muscle strength is a combination of three muscles (i.e., the TP, FDL, and flexor hallucis longus (FHL) forces. Because the FDL had a higher proportion of attachment to the posteromedial border of the tibia, which is the most common location of MTSS [12], plantar flexion strength of the lesser toes could be related to MTSS. Therefore, it was assumed that by measuring the plantar flexion strength of the hallux and lesser toes, the relationship between MTSS and muscle strength could be better understood. The relationship between MTSS and muscle strength of the hallux and lesser toes has not been evaluated due to the lack of quantitative methods.

Furthermore, MTSS is known to recur, and previous studies have reported that a patient with previous history of MTSS is at a risk of MTSS [15, 16]. This suggests that there must be some physical factor that induces MTSS in runners with a history of MTSS. Therefore, we assumed that a cross-sectional study investigating the relationships between MTSS and muscle strength of the hallux and lesser toes could be useful for estimation of MTSS risk factors. Moreover, because pain may inhibit force development, it is important to measure the muscle strength of subjects with a history of MTSS who are devoid of pain at the time of measurement.

This study aimed to conduct a detailed investigation of the muscle strength characteristics, including the muscle strength of the 1st metatarsophalangeal joint (MTPJ) and 2nd–5th MTPJ plantar flexion, in runners with a history of MTSS by using a newly developed device. The hypothesis of the study was that the strength of the 2nd–5th MTPJ plantar flexion should be higher in runners with a history of MTSS because FDL, which attaches to the most common location of MTSS, excessively functions in runners with a history of MTSS.

## Methods

### Participants

**Fig. 1:**
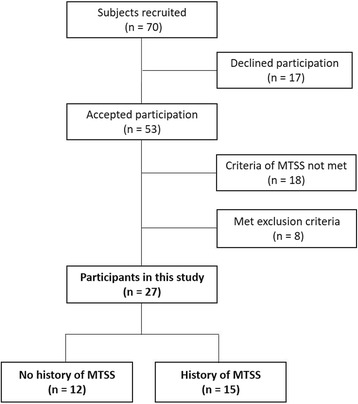
Flow diagram for participant selection Seventy runners were initially eligible for participation in this study, 17 declined to participate, 18 failed to meet the inclusion criteria of MTSS, and 8 met the exclusion criteria. Thus, 12 runners had no history of MTSS and 15 had a bilateral history of MTSS; 27 runners were included in the study

This study comprised 27 collegiate male runner participants (20.0 ± 1.6 years, 172.1 ± 5.1 cm, 57.5 ± 4.0 kg). The inclusion criteria were that the runner had a history of either bilateral or no MTSS, which was defined on the basis of the following criteria for diagnosis of MTSS: (i) exercise induced pain that is located on the posteromedial border of the tibia, (ii) palpation of the posteromedial border of the tibia that produces discomfort, and (iii) site spreading over a minimum of 5 cm, determined by reference to Yates et al. [17]. Participants with a history of unilateral MTSS, with a history of lower limb fracture and with pain in the lower limb by running at the time of intervention were excluded. A flow diagram indicating participant selection is shown in Fig. 1. Twelve participants without history of MTSS and 15 participants with a history of bilateral MTSS were included. The participants were given precise information about the content and order of the study. In addition, informed consent was obtained from all participants. This study was approved by the ethics committee of the Kyoto University Graduate School and the Faculty of Medicine (R0266).

### Procedures

#### Measurement of the foot alignment

To assess midfoot and rearfoot alignment of the participants, navicular drop and leg–heel alignment were measured. Navicular drop was calculated by subtracting the navicular height in the standing position from that in the sitting position. Navicular height was defined as the distance from the floor to the tubercle of the navicular bone, measured using a ruler [18]. Leg–heel alignment was also measured by the same observer. Leg–heel alignment was defined as the angle between the midline of the distal one-third of the leg and that of the calcaneous through a calcaneal tuberosity. Then, the foot alignment was measured by a physical therapist who had >5 years of experience in orthopedic physical therapy [19].

#### Muscle strength measurement of the ankle

Maximal voluntary isometric contraction (MVIC) torque of the plantar flexion, dorsiflexion, inversion, and eversion of the ankle were measured by using an electric dynamometer (BIODEX System 4, BIODEX, USA). Previous studies reported the reliability and validity of this instrument [20], and the intra-class correlation (ICC) of repeated measurement value of BIODEX was 0.99. Plantar flexion torque of the ankle was measured at 0° of ankle plantar-dorsiflexion and 0° of inversion–eversion. Dorsiflexion torque of the ankle was measured at 25° of ankle plantar flexion and 0° of inversion–eversion. Inversion and eversion torque of the ankle was measured at 0° of ankle plantar-dorsiflexion and 0° of inversion–eversion. The knee was at 45° flexion for all measurements. The participants were seated on the dynamometer with their trunk, upper thigh, lower thigh, and foot secured to a dynamometer by using nonelastic straps. After a warm-up session with submaximal contractions, the MVIC torques were measured for 3 s of maximal contraction for each direction while prohibiting reactive movement to determine the highest torque. The resting period was set to minimize muscle fatigue.

#### Muscle strength measurement of the MTPJ

**Fig. 2:**
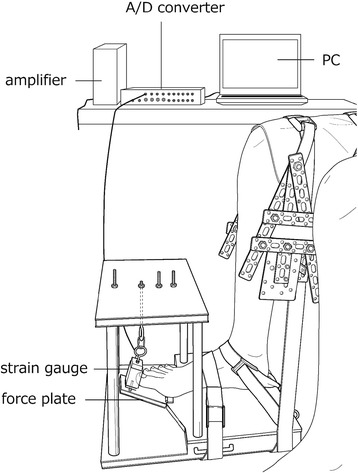
easurement of maximal isometric voluntary contraction torque of the 1st and 2nd–5th metatarsophalangeal joints

The MVIC torque values of the 1st MTPJ and 2nd–5th MTPJ were measured by using a custom-made torque-measuring device (Fig. 2). This torque-measuring device, developed by us, can quantitatively evaluate the plantar flexion torque of the 1st MTPJ and 2nd–5th MTPJ, which enabled us to evaluate the 1st and 2nd–5th MTPJ. The plantar flexion torque was measured from the tensile force of a strain gauge (TU-BR, TEAC, Japan) and the lever arm of the foot plate (0.10 m). The tensile force data induced by the plantar flexion of the MTPJ were converted from analog to digital data (Power Lab, AD instruments, Australia) via an amplifier (DPM-911B, Kyowa Electronics, Japan) and stored on a personal computer at 1000 Hz. The stored data were filtered at 10 Hz to filter low-frequency noise by using analysis software (Chart 5, AD instruments, Australia). The coefficient of variation (CV) for reliability of this strain gauge is 0.03%. The measurement device was calibrated using a luggage scale. The MVIC torque was defined as the maximal measured torque minus the resting passive torque. Plantar flexion torque values of the 1st MTPJ and 2nd–5th MTPJ were measured at 0° of ankle plantar-dorsiflexion and 30° of MTPJ. The measurement order of the toe was randomly decided. The participants were seated on a chair with their foot secured to a custom-made torque-measuring device by using nonelastic straps. After a warm-up session with submaximal contractions, each participant performed the MVIC torques of the 1st MTPJ and 2nd–5th MTPJ at each position for approximately 3 s without reaction force to determine the highest torque.

For all strength measurements, the order of measurement was quasi-randomized by participants ID. Each strength was measured twice, and a higher torque was used to determine the unilateral muscle strength. The mean value of right and left leg was used for statistical analysis.

### Statistical Analysis

Descriptive data are presented as means ± SDs. Shapiro–Wilk test were used to confirm normal distribution. To compare the foot alignment and MVIC torques between participants with and without a history of MTSS, unpaired *t*-test was used if normal distribution was confirmed, and Mann–Whitney *U* test was used if not. To assess the reliability of the muscle strength measurements in all participants (n = 27 right legs) in this study, ICC (1.1) was determined. In addition, CV was assessed by dividing the standard deviation of two repeated measurements by the average of two measurement values. Statistical analyses were performed by using statistical software (SPSS Statistics 22, IBM, USA). For all tests, statistical significance was set at *p* < 0.05.

## Results

Normal distribution was confirmed in all measurements of alignment and strength except for leg–heel alignment and inversion strength of the ankle. The results of the foot alignment are shown in Table 1. For both navicular drop and leg–heel alignment, there was no significant difference between participants with and without a history of MTSS. (*p* = 0.98 for navicular drop and 0.72 for leg–heel alignment). The reliability results are shown in Table 2, and the results of the strength are shown in Table 3. For the 1st MTPJ plantar flexion, the MVIC torque was 9.8 ± 2.3 N∙m in the participants without history of MTSS and 12.0 ± 3.0 N∙m in the participants with a history of MTSS, with significant difference (*p* = 0.04). For the 2nd–5th MTPJ plantar flexion, the MVIC torque was 6.0 ± 1.6 N∙m in the participants without history of MTSS and 6.0 ± 2.1 N∙m in the participants with a history of MTSS, but the difference was not significant (*p* = 0.94). For the MVIC torque of the ankle, there was no significant difference between participants with and without a history of MTSS (*p* = 0.45 for plantar flexion, 0.75 for dorsi flexion, 0.66 for eversion and 0.90 for inversion).

**Table 1:** Foot alignment in participants with and without a history of MTSS

	No history of MTSS	History of MTSS	P-value	Effect size (r)
Navicular Drop (mm)	7.1 ± 2.0	7.1 ± 1.9	0.98	0.01
Leg Heel Alignment (°)	5.0 ± 1.4	5.1 ± 2.0	0.72	0.08

*MTSS*, medial tibial stress syndrome

**Table 2:** Reliability values of the muscle strength measurements

Joint	Direction	ICC (1.1)	CV (%)
Ankle	Plantar flexion	0.93	7.3
	Dorsiflexion	0.83	6.8
	Eversion	0.86	9.2
	Inversion	0.64	9.8
1st MTPJ	Plantar flexion	0.96	4.8
2nd–5th MTPJ	Plantar flexion	0.94	8.3

Intra-class correlation (ICC) (1.1) was calculated using two intra-day measurements of the muscle strength in this study. In addition, the coefficient of variation (CV) was calculated. CV was calculated by dividing the standard deviation of two repeated measurements by the average of two measurement values

**Table 3:** Muscle strengths of the ankle and toe in participants with and without a history of MTSS

Joint	Direction	MVIC torque (N·m)		P-value	Effect size (r)
		No history of MTSS	History of MTSS		
Ankle	Plantar flexion	84.2 ± 28.2	92.2 ± 28.2	0.45	0.15
	Dorsiflexion	32.7 ± 5.4	33.3 ± 5.3	0.75	0.07
	Eversion	21.6 ± 5.7	22.6 ± 5.8	0.66	0.09
	Inversion	23.5 ± 9.9	22.0 ± 5.9	0.90	0.02
1st MTPJ	Plantar flexion	9.8 ± 2.3	12.0 ± 3.0	0.04	0.40
2nd–5th MTPJ	Plantar flexion	6.0 ± 1.6	6.0 ± 2.1	0.94	0.02

*MTSS*, medial tibial stress syndrome

*MTPJ*, metatarsophalangeal joint

*MVIC*, maximal voluntary isometric contraction

## Discussion

This study investigated muscle strength characteristics in runners with a history of MTSS by comparing the muscle strength of the ankle and toe between runners with and without a history of MTSS. To the best of our knowledge, this is the first study to show the relationship between MTSS and toe strength. For both navicular drop and leg–heel alignment, there was no significant difference between participants with and without a history of MTSS. Thus, the foot alignment of participants was not different between the two groups. The ICC values for all muscle strength measurements were >0.61, and substantial reliability was confirmed [21].

In this study, the MVIC torque of the 1st MTPJ plantar flexion was significantly higher in runners with a history of MTSS than without it. In contrast, there was no significant difference in MVIC torque of the 2nd–5th MTPJ plantar flexion between the runners with a history of MTSS and without it. These results did not support our hypothesis that MTSS occurs because of excessive activity of the FDL, which could be identified as higher MVIC torque of the 2nd–5th MTPJ plantar flexion in runners with a history of MTSS. There are two possibilities regarding these results.

Our results suggest that runners with a history of MTSS adopt a strategy of reducing the load to the medial tibia because of their history of MTSS. A previous study reported that excessive pronation during motion is a risk factor of developing MTSS [9, 11]. The results also suggest that the FDL and TP muscles that act to support the arch of the foot tend to be stressed in runners who potentially have a risk for developing MTSS. On the other hand, although the FHL, which is an agonist of 1st MTPJ plantar flexion, has a function similar to those of the FDL and TP as the inversion muscle of the ankle, FHL is not likely to be related to development of MTSS because the FHL does not connect to tibial fascia [22]. Thus, this characteristic increase in MVIC torque of the 1st MTPJ in runners with a history of MTSS could be considered to be a result of increased activity of the FHL to avoid pain caused by contraction stress of the FDL, which could be a possible cause of MTSS. Although the activity of intrinsic and extrinsic muscles cannot be distinguished by measuring the MTPJ plantar flexion strength, our previous study investigated the associations between navicular drop and toe flexion strength at a different angle of the ankle and toe, and the result showed that navicular drop was correlated with only toe flexion strength at the position where the intrinsic foot muscles are relatively elongated [23]. This previous study indicated the correlation between intrinsic foot muscle strength and navicular drop. In the current study, navicular drop was not different between the two groups. Therefore, we considered that the activity of intrinsic muscle was not different between the groups while measuring the MTPJ plantar flexion strength.

Surprisingly, there was no significant difference in MVIC torque of the 2nd–5th MTPJ plantar flexion between runners with and without a history of MTSS. If runners with a history of MTSS adopt a strategy of reducing activation of the FDL by relying on the FHL to avoid pain during running, the MVIC torques of the 2nd–5th MTPJ could be lower. A previous study reported that the FHL tendon branches to the 2nd and 3rd toe in most cases [24]. Therefore, it could be considered that the absence of a significant difference in the MVIC torques of the 2nd–5th MTPJ between runners with and without a history of MTSS could be due to the effects of the branching of FHL tendon to the 2nd and 3rd toe. Collectively, muscle strength characteristics in runners with a history of MTSS could be considered to be a result of increasing activity of the FHL to reduce the load on FDL and avoid pain caused by contraction stress of the FDL.

There were no significant differences in the MVIC torque values at the ankle between runners with and without a history of MTSS. This result supports a previous study showing that the isometric strength of the ankle measured in painless legs was not related to the development of MTSS [15]. Collectively, these results suggest that characteristics in runners with a history of MTSS can be best understood by measuring toe strength rather than the ankle strength.

There are a few limitations in this study. First, the sample size was not enough in this study. Although the sample size was insufficient, we considered that the results were important because at least a moderate effect size was observed in the 1st MTPJ plantar flexion. Second, we investigated the relationships between MTSS and muscle strength as a static assessment. Therefore, it is unclear whether the results directly reflect muscle endurance or activity during running. However, in this study, measurement position of the ankle and MTPJ is same as the angle when the highest MTPJ moment is produced during running [25, 26]. Thus, it is considered that the MTPJ plantar flexion strength at this angle represents a certain aspect during running. Future study is needed to investigate the associations between MVIC torques in the MTPJ plantar flexion and endurance or dynamic assessments (e.g., alignment during running and plantar pressure of the foot).

## Conclusions

This study investigated the muscle strength characteristics in runners with a history of MTSS by comparing the muscle strength of the ankle and toe between runners with and without a history of MTSS. The results showed that a history of MTSS increased the isometric FHL strength.

## Abbreviations

FDL: Flexor digitorum longus
FHL: Flexor hallucis longus
MTPJ: Metatarsophalangeal joint
MTSS: Medial tibial stress syndrome
MVIC: Maximal voluntary isometric contraction
SOL: Soleus
TP: Tibialis posterior
